# Mucosal IgA and IFN-γ^+^ CD8 T cell immunity are important in the efficacy of live *Salmonella enteria* serovar Choleraesuis vaccines

**DOI:** 10.1038/srep46408

**Published:** 2017-04-13

**Authors:** Liangquan Zhu, Xinxin Zhao, Qing Yin, Xianyong Liu, Xiang Chen, Chunjuan Huang, Xun Suo

**Affiliations:** 1 Key Laboratory of Animal Epidemiology and Zoonosis of Ministry of Agriculture, State Key Laboratory for Agrobiotechnology, National Animal Protozoa Laboratory & College of Veterinary Medicine, China Agricultural University, Beijing, China; 2 China Institute of Veterinary Drug Control, Beijing, China; 3 Institute of Preventive Veterinary Medicine, Sichuan Agricultural University, Chengdu, Sichuan, China; 4 Jiangsu Key Laboratory of Zoonosis, Yangzhou University, Yangzhou, Jiangsu, China; 5 Institute of Biophysics, Chinese Academy of Sciences, Beijing, China

## Abstract

Salmonellosis, a disease caused by non-typhoidal *Salmonella* strains which can be transmitted from swine to humans, is one of the leading public health problems around the world. Paratyphoid of swine is controlled by vaccinating swine with *Salmonella enterica* serovar Choleraesuis (*S.* Choleraesuis) live vaccine strain C500 in China. Although the vaccine has good prophylactic efficacy, the mechanism of immunogenicity is unclear. Using a C500-derived paratyphoid thermo-stable live vaccine (PTSL vaccine), we demonstrated that the PTSL vaccine induces strong primary and memory immune responses in piglets. Mucosal IgA and IFN-γ^+^/CD8^+^ T cells induced by the PTSL vaccine play key roles in the protection of the host from *Salmonella* infection. Our findings have important implications on the development of new and improved vaccines against salmonellosis and using live-attenuated *Salmonella* as vaccine carriers.

*Salmonella*, which is transmitted to humans via contaminated food and water, is the second most common bacterium causing food-borne gastroenteritis in humans^1^. *Salmonella enterica* serovar Choleraesuis (*S.* Choleraesuis) mainly causes swine paratyphoid in weaners younger than 4 months old^2^, and can also cause food-borne diseases in humans^3^,^4^,^5^. Septicemia is the typical acute clinical syndrome leading to death of piglets. However, most infected animals develop chronic infections characterized by wasting syndromes, such as malaise, anorexia, pyrexia, dyspnea, and pneumonia^6^,^7^. Sub-clinically infected animals continuously shed pathogenic bacteria into the environment, resulting in environment contamination and zoonosis^2^,^8^.

Vaccination is an effective tool to prevent *Salmonella* infection and spreading. The licensed attenuated *S.* Choleraesuis vaccine, strain C500, is safe, and has been used to prevent swine paratyphoid in China for the past 40 years^9^,^10^. As a facultative intracellular bacterium, the vaccine strain has also been studied as an oral vaccine vector to deliver heterologous protein or DNA vaccine, which provides protection against infectious diseases in addition to paratyphoid^11^,^12^.

However, the type and magnitude of adaptive immune responses induced by the *S.* Choleraesuis C500 vaccine, and the protective mechanism have not yet been elucidated. A better understanding of the immune response of the host to *Salmonella* can provide a solid foundation for the development of vaccines against salmonellosis and recombinant vaccines using *Salmonella* as the vector against other infectious diseases. *Salmonella* infection and adaptive immunity have been extensively studied in mouse models in laboratories by scientists^11^,^13^,^14^,^15^,^16^, but the protective immunity against paratyphoid in piglets has not been well characterized despite widespread infection of *Salmonella* in piglets.

We developed a paratyphoid thermo-stable live vaccine (PTSL vaccine), derived from the C500 strain by adding the thermo-tolerant stabilizer during the manufacturing process^17^. Compared to the C500 vaccine, the PTSL vaccine has an increased survival ratio of more than 80% during lyopholization and prolonged storage period of 24 months at 2–8 °C^18^. In this study, we investigated the immune responses and protective efficacy of the PTSL vaccine in piglets in the field. In addition, we further analyzed the correlation between the protective efficacy and the elicited immune responses.

## Results

### The PTSL vaccine elicited strong antibody responses

**Figure 1:**
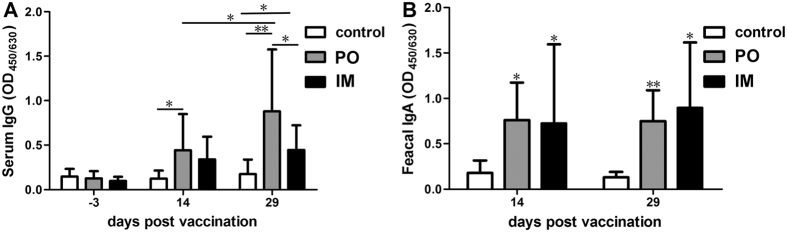
The PTSL vaccine elicited high levels of serum (**A**) and mucosal (**B**) antibody responses in piglets (n = 20) by both PO and IM vaccination. Serum IgG and fecal (anal swab) IgA were determined by indirect ELISA. *p < 0.05; **p < 0.01.

Serum anti-*S.* Choleraesuis IgG was measured in piglets of non-vaccinated control group (control), oral vaccination group (PO) and intramuscular vaccination group (IM). As shown in Fig. 1A, the *S.* Choleraesuis specific IgG level in the piglets of vaccinated groups increased significantly with time after vaccination, compared with negligible IgG in piglets of all the three groups before vaccination. The IgG level in piglets of the PO group was higher than that in the IM group. We also compared the IgG responses in piglets immunized with 1/2× or 2× the PTSL vaccine dose. The IgG level in piglets with IM immunization increased with increasing vaccine dosage, indicating that the magnitude of serum IgG response was dependent on the dosage by the IM route. On the other hand, the IgG level in the piglets vaccinated by the PO route was dose-dependent at the vaccine dosages of 1/2× and 1× dose, but there was no difference between the dosages of 1 and 2× the nominal vaccine dose (Fig. S1). Higher IgG levels were detected in piglets of the PO group than the IM group at the vaccine dosages of 1/2× and 1× dose, and this was reversed at the dosage of 2× the nominal dose (Fig. S1). Therefore, the magnitude of humoral response was dependent on both the immunization route and dosage. The PTSL vaccine also induced specific fecal IgA in piglets of both the PO and IM groups on the 14^th^ and 29^th^ day post vaccination (dpv), and the fecal IgA responses were similar in the PO and IM groups (Fig. 1B). Thus, the PTSL vaccine induces both humoral and mucosal antibody responses in pigs.

### The PTSL vaccine elicited Th1 type T-cell immune responses

**Figure 2:**
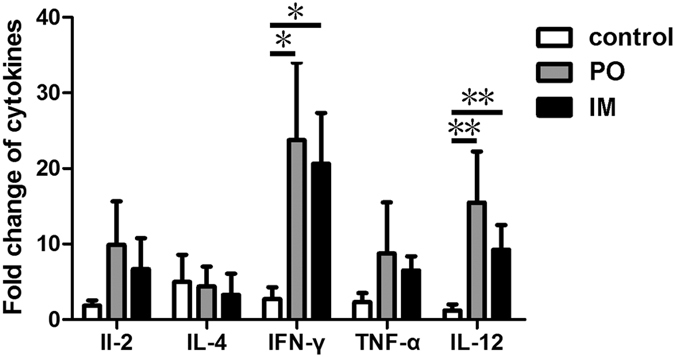
The PTSL vaccine elicited Th1 type cell-mediated immune responses (i.e. T cells expressing IL-2, IFN-γ and TNF-α) in piglets. PBMCs were isolated from five piglets of the control, PO and IM groups on the 29^th^ day post vaccination (dpv), and were stimulated with inactivated *S.* Choleraesuis antigen *in vitro*. RNA was extracted for determination of IL-2, IL-4, IL-12, IFN-γ and TNF-α expression levels by RT-PCR. The results are presented as fold of expression in unstimulated control cells from each animal. *p < 0.05; **p < 0.01.

To characterize the T-cell immune response induced by the PTSL vaccine, the profile of cytokine expression in porcine PBMCs stimulated by inactivated *S.* Choleraesuis antigen was determined by RT-PCR. As shown in Fig. 2, IL-2, IFN-γ, and TNF-α, the pro-inflammatory cytokines associated with a Th1-type immune response^19^, were significantly up-regulated, up to about 10 folds higher than un-stimulated cells, from vaccinated piglets. The up-regulated IL-12 was also related to the Th1 type T-cell immune response, as IL-12 promotes the proliferation of activated T cells and the differentiation of Th0 into Th1 cells^20^. However, the transcriptional level of IL-4, a Th2 type cytokine, remained unchanged. Among the up-regulated Th1 type cytokines, IFN-γ had the highest increase of about 20 fold in the vaccinated groups compared to the control group. In addition, the levels of IL-2, IFN-γ and TNF-α were higher in piglets of the PO group than in the IM group. The findings indicated that the PTSL vaccine elicited Th1 type cell-mediated immune responses, and the PO route induced stronger Th1 type immune responses than the IM route.

### The PTSL vaccine induced the expression of IFN-γ by CD4^+^ and CD8^+^ T cells

**Figure 3:**
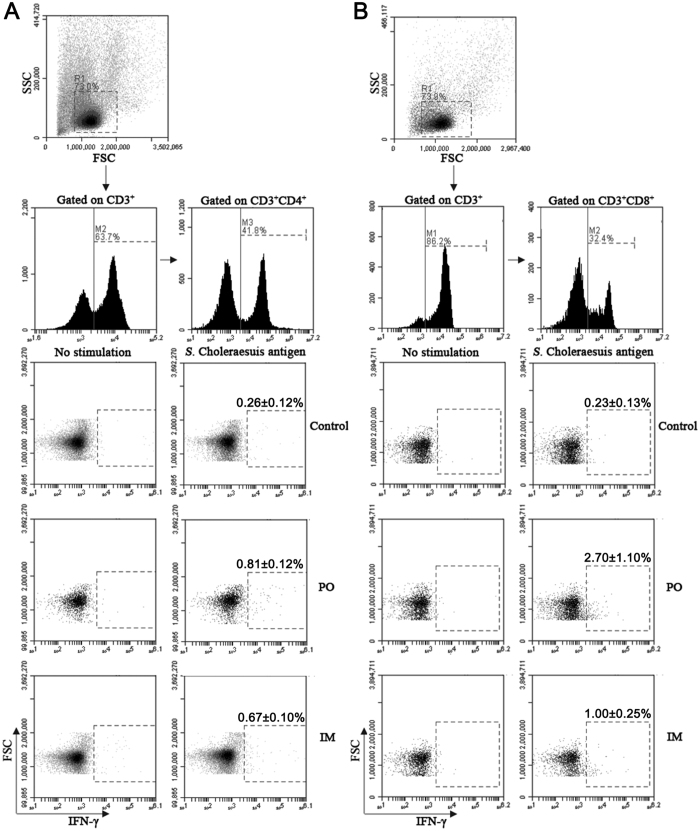
The PTSL vaccine induced IFN-γ^+^ expression in CD4^+^ and CD8^+^ T cells. PBMCs were isolated from 5 piglets on the 14^th^ dpv, and then stimulated with inactivated *S.* Choleraesuis antigen *in vitro*. The cells were stained with appropriate anti-pig antibodies before flow cytometry analyses for IFN-γ production in CD4^+^/CD3^+^ T cells (**A**) and CD8^+^/CD3^+^ T cells (**B**). *p < 0.05.

IFN-γ plays an important role in the defense against intracellular pathogens, such as *Salmonella*^21^. The frequencies of *S.* Choleraesuis-specific IFN-γ^+^ CD4^+^ and IFN-γ^+^ CD8^+^ T cells in porcine PBMCs were determined on the 14^th^ dpv. As shown in Fig. 3A, approximately 0.81% and 0.67% of the IFN-γ^+^ CD4^+^ T cells were detected in piglets of the PO and IM groups, respectively, compared with 0.26% IFN-γ^+^ CD4^+^ T cells in the control group. The frequency of IFN-γ^+^ CD8^+^ T cells also increased in the immunized groups compared to the control group, and the frequency in the PO group was higher than in the IM group (approximately 2.7%, 1.0% and 0.23% in the PO, IM and control groups, respectively, Fig. 3B). Thus, the vaccine induced IFN-γ in both CD4^+^ and CD8^+^ T cells, and IFN-γ expressed in CD8^+^ T was higher in the piglets of the PO group than in the IM group.

### The PTSL vaccine protected piglets from virulent challenge

**Figure 4:**
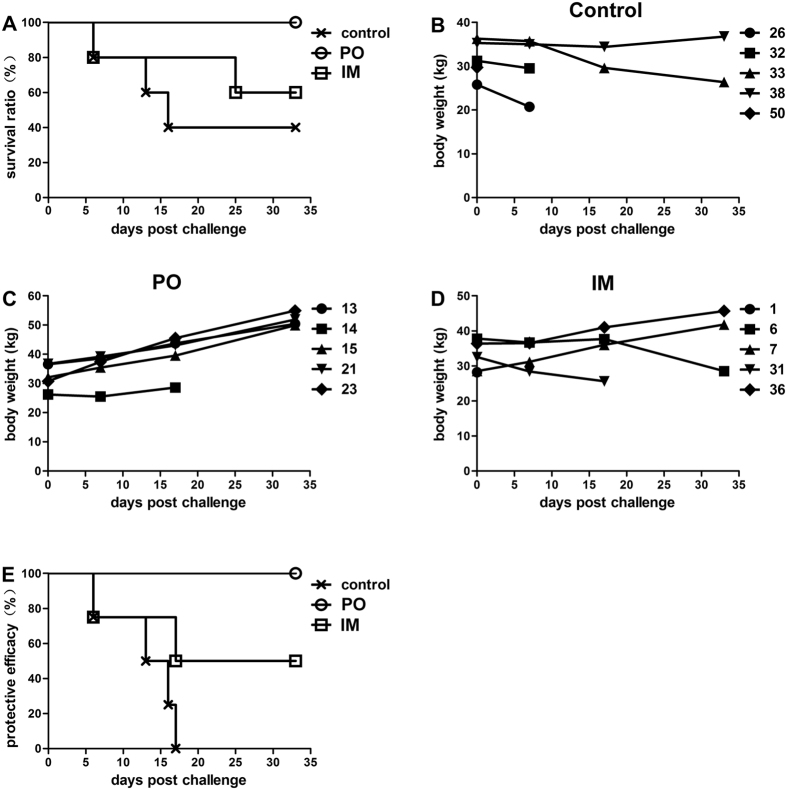
The PTSL vaccine protected piglets from virulent *S.* Choleraesuis challenge. Five randomly selected piglets from each group were challenged with 3.0 × 10^8^ CFU C78-2 i.v. (**A**) survival data; (**B–D**) individual piglet body weights of the control (**B**), PO (**C**), and IM (**D**) groups. (**E**) protective efficacy (surviving animals with body weight gain were considered to be protected by vaccination).

To evaluate the protective potency of the vaccine, the mortality and body weight of piglets were recorded post challenge. Three piglets of the control group died post challenge (Table 1 and Fig. 4A), and they exhibited symptoms of septicemia or enteritis along with trembling and diarrhea (data not shown). Two piglets of the IM group died, but all five piglets of the PO group remained alive post challenge. *S.* Choleraesuis was isolated from the solid organs of all the dead piglets except one of the IM group (No. 31), indicating that this piglet’s death was not caused by the C78–2 strain challenge and thus excluded from data analysis. Furthermore, the remaining two surviving piglets of the control group (No. 33 and No. 38) and one surviving piglet of the IM group (No. 6) showed growth retardation post challenge (Table 1 and Fig. 4B and D). In contrast, two piglets of the IM group and all the piglets of the PO group steadily gained weight post challenge (Fig. 4C and D). The protective efficacy (number of protected animals/number of challenged animals) was 100% (5/5), and 50% (2/4) by the PO and IM route, respectively (Fig. 4E). The PTSL vaccine protected vaccinated piglets from virulent *S.* Choleraesuis challenge, and the PO route conferred better protection than the IM route.

**Figure 5:**
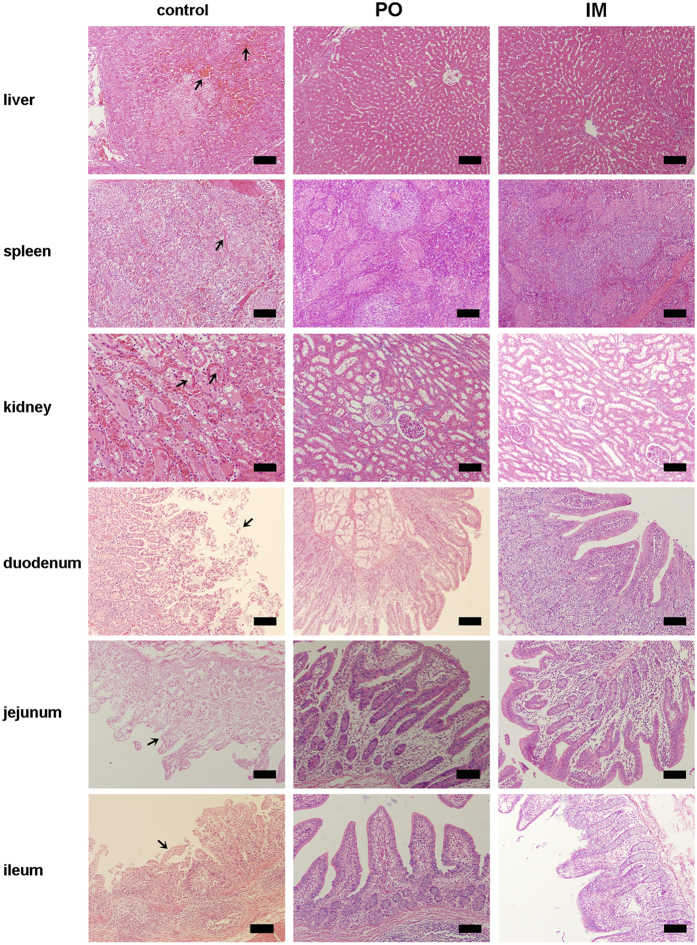
Micrographs of liver, spleen, kidney and small intestines of challenged piglets immunized PO or IM with the PTSL vaccine or non-vaccinated control piglets. The challenged, non-vaccinated control animals (left panel) developed severe lesions in all the tissues: sinusoidal congestion, hepatocellular necrosis and hemorrhage of liver; hemorrhage and disappearance of white pulp of spleen; tubular epithelial cell necrosis and sloughing of epithelial cells of kidney; exfoliation of mucosal epithelium of duodenum and jejunum; and disintegration of ileum epithelium. The lesions are marked with an arrow. Tissues of the IM and PO immunized piglets appeared normal (middle and right panels). Bar, 200 μm.

Gross pathology and histopathology examination was carried out to investigate the effect of the virulent *S.* Choleraesuis strain in the piglets (Fig. 5). Severe lesions in the liver, spleen, kidney and small intestines were observed in the dead piglets of the non-vaccinated control group. Liver showed sinusoidal congestion, hemorrhage, and hepatocellular necrosis. Splenic hemorrhage and disappearance of white pulp were evident in spleen, and tubular epithelial cell necrosis and epithelial cell sloughing in kidneys. The mucosa of duodenum, jejunum, and ileum had typical exfoliation. No pathological lesions were observed in the vaccinated groups, and the organs and tissues of these piglets had a normal appearance. The results indicate that the PTSL vaccine could protect piglets from paratyphoid disease, as the immunized piglets did not develop any clinical signs of the disease or pathological lesions after challenging with a virulent *Salmonella* strain.

### Potent memory immune responses were induced by virulent challenge

**Figure 6:**
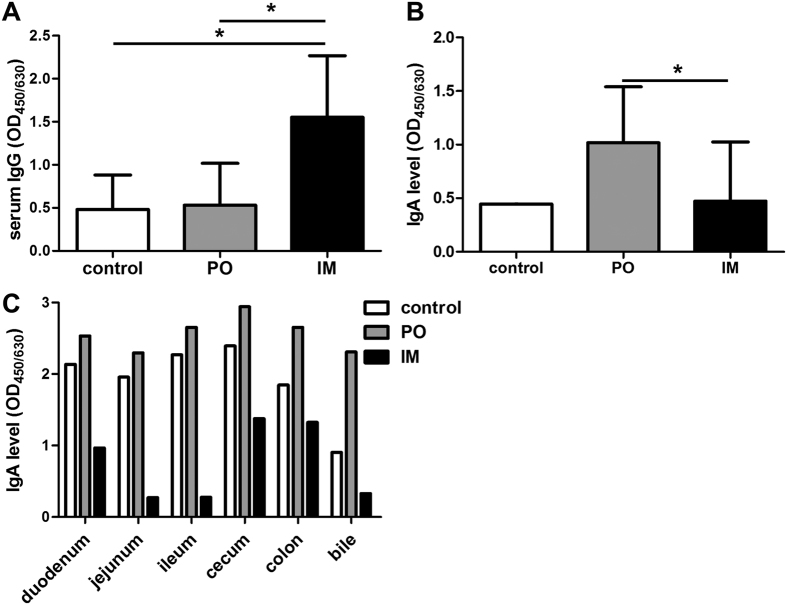
Memory humoral and mucosal immune responses were induced in piglets immunized with the PTSL vaccine. Sera were collected from three randomly selected surviving piglets from each group on the 8^th^ dpc (n = 3). Anal swabs were collected on the 24^th^ dpc, from the two surviving piglets of the unvaccinated control group (n = 2), three randomly selected piglets of the PO and IM group (n = 3). Bile and intestines were collected from the two surviving piglets of the unvaccinated control group, two randomly selected piglets of the PO group, and the two protected piglets of the IM group (n = 2), and processed at necropsy on the 33^rd^ dpc. IgG in serum (**A**), fecal IgA (**B**) and IgA in bile and intestinal mucosa (**C**) were determined by indirect ELISA. *p < 0.05, **p < 0.01.

Memory IgG responses were determined on the 8^th^ day post challenge (dpc). The IM vaccinated piglets exhibited a high memory serum IgG response to the challenge compared with the unvaccinated and the PO vaccinated piglets (Fig. 6A). At the 24^th^ dpc, the PO vaccinated piglets showed a high level of memory fecal IgA response, compared with levels in the unvaccinated and IM vaccinated piglets (Fig. 6B). Mucosal IgA memory responses of challenged piglets were assessed by bile and intestinal IgA levels. In contrast to the recall IgG response, the recall IgA response of piglets in PO group was much higher than that of piglets in IM groups, while the IgA level in unvaccinated control varied from 40–80% of that in the PO group (Fig. 6C).

**Figure 7:**
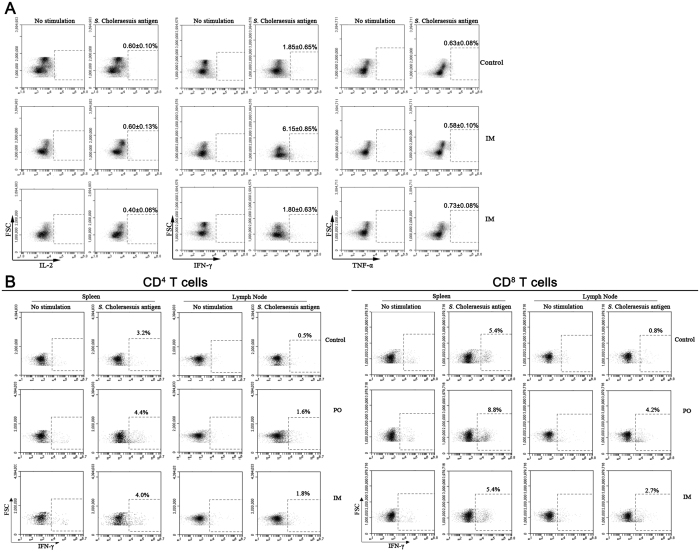
Memory cell-mediated immune response was induced in piglets immunized with the PTSL vaccine. PBMCs were collected from three protected piglets from each group on the 18^th^ dpc (n = 3). Spleen and mesenteric lymph node (MLN) were collected from the two surviving piglets of the unvaccinated control group, two randomly selected piglets of the PO group, and the two protected piglets of the IM group (n = 2), and processed at necropsy on the 33^th^ dpc. IL-2, IFN-γ or TNF-α positive T cells (CD3^+^) in PBMCs (**A**) were measured by flow cytometry after stimulation with inactivated *S.* Choleraesuis antigen and staining with labelled anti-pig antibodies. IFN-γ positive CD4^+^ and CD8^+^ T cells in spleen and MLN (**B**) were also measured.

Memory T cell responses in challenged piglets were assessed by intracellular cytokine staining. As shown in Fig. 7A, approximately 0.4–0.6% IL-2^+^ T cells and 0.6–0.7% TNF-α^+^ T cells were detected in the piglets of the unvaccinated control, PO and IM groups, with no significant difference among groups. Interestingly, approximately 6.1% IFN-γ^+^ T cells were detected in piglets of the PO group, while just 1.8% IFN-γ^+^ T cells in the unvaccinated control and IM groups. The results indicated that *S.* Choleraesuis-specific T cells mainly produced IFN-γ. In addition, the IFN-γ production was assessed in CD4^+^ and CD8^+^ T cells obtained from the spleen and mesenteric lymph node (MLN) of piglets in the control, PO and IM groups. Approximately 3.2–4.4% of IFN-γ^+^/CD4^+^ T cells were detected in the spleen in all three groups. The proportion of IFN-γ^+^ CD4^+^ T cells in MLN was about 1.6–1.8% in the PO and IM groups, about 3-fold higher than that in the control group (Fig. 7B). The proportion of IFN-γ^+^/CD8^+^ T cells in both spleen (8.8%) and MLN (4.2%) was much higher in the piglets of the PO group than in the control and IM groups (Fig. 7B). Thus, potent memory humoral and T-cell responses were elicited in the vaccinated piglets by the virulent challenge. Compared to the IM group, the PO group generated stronger mucosal IgA response, and more IFN-γ^+^ was produced in CD8^+^ T cells. This was consistent with the better protective efficacy exhibited in the PO group than the IM group, indicating that mucosal IgA and IFN-γ^+^/CD8^+^ T cells play important roles in the protection of piglets against salmonella infection.

## Discussion

The PTSL vaccine is a thermo-stable vaccine derived from C500. This was the first study investigating the immune responses and protective efficacy of the PTSL vaccine in its native host, swine. We showed that the PTSL vaccine elicited primary and memory immune responses, encompassing humoral, cellular and mucosal immunity. Vaccination by the oral route protected piglets against a virulent challenge, correlated with high expression of mucosal IgA and IFN-γ^+^/CD8^+^ T cells.

Broad adaptive immune responses were stimulated by a single PO or IM dose of the PTSL vaccine. Vaccination induced high *S.* Choleraesuis specific serum IgG levels, blood IFN-γ^+^/CD4^+^ and IFN-γ^+^/CD8^+^ T cell populations, and mucosal IgA, which was consistent with previous reports of *S.* Choleraesuis vaccine strain SC-54, *S.* Choleraesuis Δcrp/vpl^−^ 8,15,22. This indicated that after vaccination via the PO or IM route, live *S.* Choleraesuis or *Salmonella* antigen can reach both the mucosal immune organs, i.e. Peyer’s patches, like the internalization of SC-54 into Peyer’s patches^23^, and MLN) and peripheral immune organs, such as the spleen. A higher proportion of the effector CD8^+^ T cells were induced than CD4^+^ T cells, consistent with observations in mice in response to infection reported by Foulds and co-authors^24^, who showed restricted proliferation of CD4 T cells compared with extensive proliferation of CD8 T cells in response to bacterial infection.

The PTSL vaccine elicited a Th1 cell-mediated response, as indicated by the significant up-regulation of hallmark Th1 cytokines, IL-2, IFN-γ, and TNF-α in T cells, also IL-12, the facilitator of Th1-type T cell immune response. This observation is consistent with previous studies with *S.* Choleraesuis^16^, *Salmonella* Typhimurium^25^,^26^, and *Salmonella* Typhi^27^. However, the magnitude of immune responses induced by the PO and IM immunization routes was different. The response of IFN-γ^+^ CD4^+^ T cells was similar by PO or IM immunization, but the IFN-γ^+^ CD8^+^ T cell response to the vaccine by PO administration was nearly three times that by the IM route. Compared to the CD4^+^ T cell response, a longer duration was required by an antigen to initiate CD8^+^ T cell response^28^,^29^. Thus we speculated that the PTSL strain had undergone better colonization and proliferation through oral inoculation, resulting in more antigens reaching the spleen and MLN, and remained there for a longer duration. In addition, we found that serum IgG response induced by IM immunization was dependent on the dosage of the vaccine from half to twice the standard vaccine dose, while there was no difference in IgG level at oral vaccination doses of 1× and 2× the standard dose, which was higher than the IgG level after vaccination with half the standard oral dose (Figure S1). Thus, the recommended oral dose of the PTSL vaccine is appropriate and induces optimal humoral immune responses in pigs. Our study proved that the PTSL vaccine is highly immunogenic and effective against Salmonella infection.

It is important that vaccines induce potent long-lasting memory immunity^30^,^31^. The PTSL vaccine by PO administration stimulated adequate memory responses, including mucosal and peripheral humoral immunity and T-cell mediated immunity, and protected piglets against a virulent challenge. Different from the oral vaccination, the IM vaccination induced only weak mucosal and cellular immune responses to the virulent challenge as shown by low fecal IgA, mucosal IgA in the intestine and bile and low IFN-γ^+^/CD8^+^cytotoxic T cell populations in spleen and mesenteric lymph node, compared to the oral route, although the IFN-γ^+^/CD4^+^ population was comparable between the PO and IM routes of vaccination. The comparatively low memory responses in IM vaccinated piglets correlated with low survival of the IM group challenged with a virulent dose of *Salmonella* bacteria. Our findings are consistent with previous reports that both antibody and cell-mediated responses (both CD4^+^ and CD8^+^ T cells) are required for preventing from *Salmonella* infection^14^,^25^,^32^,^33^.

A previous study showed that mucosal IgA and CD^4+^/CD8^+^ T cells played important roles in the protective efficacy of *S.* Choleraesuis vaccine^15^. Our results showed that complete protective immunity can be provided only through high mucosal IgA response along with significant response of IFN-γ production by CD8^+^ T cells. The presence of *Salmonella*-specific mucosal IgA serves to rapidly deliver the antigen to Peyer’s patch dendritic cells and also rapidly activate the adaptive immunity during secondary exposure^34^,^35^,^36^. Th1 cytokines, especially IFN-γ was considered to play a critical role in controlling human salmonellosis^21^. Our study suggested that IFN-γ is an important cytokine in pigs in immunity against *Salmonella* infection. CD8^+^ T cells appeared to be the major source of IFN-γ in swine, since the frequency of IFN-γ^+^ CD8^+^ T cell was approximately 2–3.5 time that of IFN-γ^+^ CD4^+^ T cells after immunization and challenge by oral immunization with the PTSL vaccine. Since immune responses differ in different hosts, we proposed that studies in the native host are indispensable for the illustration of immune responses to *Salmonella* infection. Our findings demonstrated that mucosal IgA and IFN-γ^+^ CD8^+^ T cells play an important role in protective immunity against virulent *S.* Choleraesuis infection in pigs. Thus, both the mucosal and cellular immunity should be targeted for developing vaccines against paratyphoid in pigs.

As the oral route is the natural means of *Salmonella* infection, oral challenge is desirable to mimic natural infection. In our study, we used systemic challenge by i.v. injection in order to rapidly induce acute infection to mimic the manifestation of severe infection, and septicemia of paratyphoid. When carried out in the laboratory, the PTSL vaccine by oral immunization provided 100% protection, compared with the efficacy of 80% of the traditional C500 vaccine^18^.

This study provides a better understanding of the primary and memory immune responses to the PTSL vaccine, as well as the protective mechanism. In particular, the oral administration strategy proved to be more effective for live attenuated *Salmonella* vaccine. The observations of our study have important implications on the development of new and improved vaccines against salmonellosis in animal species and using live-attenuated *Salmonella* as vaccine carriers.

## Materials and Methods

### *S.* Choleraesuis

*S.* Choleraesuis strain C500 was chosen for the preparation of the PTSL vaccine^9^,^10^. *S.* Choleraesuis strain C500 master seeds were grown at 37 °C in a nutrient broth medium (Zhonghai, Beijing, China) for 18 h. Then, the harvested culture was centrifuged at 3,500 rpm for 15 min at room temperature, and the pellets were suspended in a thermo-tolerant stabilizer (Zhonghai) to a final concentration of 500 × 10^8^ CFU/ml^17^. Next, every 2.6 ml of the suspension was sub-packaged into 7.0 ml vials and freeze-dried in a GMP manufacture facility to prepare the final product (LvDu, Shandong, China). Thus, the PTSL vaccine in each vial contained 30 doses, with each dose containing approximately 38.4 × 10^8^ CFU of live *S.* Choleraesuis bacteria.

*S.* Choleraesuis strain C78–2, which was a domestic virulent strain isolated from Jiangsu Province, China and kindly donated by the China Veterinary Culture Collection Center was used as the challenge strain^9^. The bacteria were revived from lyophilization on nutrient broth agar (Zhonghai). After incubation at 37 °C for 18–24 h, five typically round, humid, and smooth colonies were selected and inoculated into 2 ml Beef Liver Gastric Digest Broth (Zhonghai). After 18 h of static incubation at 37 °C, 1 ml broth was diluted in 50 ml of the same culture broth. After another 18 h of static incubation at 37 °C, the culture was harvested in C78-2 to a bacteria count of 22 × 10^8^ CFU/ml, and then diluted to 1.5 × 10^8^ CFU/ml using saline.

### Immunization and challenge

The study was conducted in a pig farm of Binzhou Shuhongchengxin Breeding Company of Shandong province in China. The study was approved by the laboratory Animal Ethics Committee of China Agricultural University and China Institute of Veterinary Drug Control, and also permitted by the Ministry of Agriculture and Bureau of Animal Husbandry and Veterinary Medicines of Shandong Province. The experiments were performed in compliance with “Regulations of the People’s Republic of China on the Administration of Experimental Animals”, “Measures of Shandong province for the administration of experimental animals” and “Guidelines for the ethical review of experimental animal welfare in Beijing”. Sixty early weaning piglets (Domestic White × Landrace) at the age of 30 days and body weight of approximately 7–8 kg were selected for immunization. The selected piglets were confirmed to be seronegative for *Salmonella* by ELISA. Every 5 piglets of the same treatment group were housed in one individual pen. Feed and water were free of antibiotics from one week before, and throughout the whole study period. Animal care during the study was conducted in accordance with the above guidelines.

The piglets were randomly divided into three groups (n = 20/group) as control, PO and IM. The PO group was vaccinated with one oral dose of the PTSL vaccine in 5 ml of 0.85% physiological saline solution using a 12-gauge ball-tipped gavage syringe. The IM group received a single dose of the vaccine in 1 ml of 20% aluminum hydroxide gel saline solution (LvDu) by intramuscular injection into the posterior ear muscles. The piglets of the control group received no vaccine.

According to the animal welfare and minimum usage guidelines^37^,^38^, five randomly selected piglets from each group were subjected to virulent challenge by intravenous (i.v.) injection with live C78-2 bacteria the minimal lethal dose of 3.0 × 10^8^ CFU^38^ in 2 ml saline on the 30^th^ dpv. All the challenged piglets were housed in a negative pressure pen at ShanDong LvDu Bioscience & Technology Co., Ltd, China. The morbidity and mortality were observed and recorded every day for 33 days post challenge^38^. The body weight was measured on the 7^th^, 17^th^ and 33^th^ dpc. All surviving piglets were sacrificed by euthanasia on the 33^th^ dpc and subjected to necropsy. Any piglet that died before the 33^th^ dpc was necropsied as soon as possible for the isolation and characterization of *S.* Choleraesuis in organs including liver, lung, spleen and kidney^39^. The carcasses of dead piglets were disposed of according to the Chinese Standard GB16548 - Biosafety Specification on Sick Animal and Animal Product.

### Sample collection

**Figure 8:**
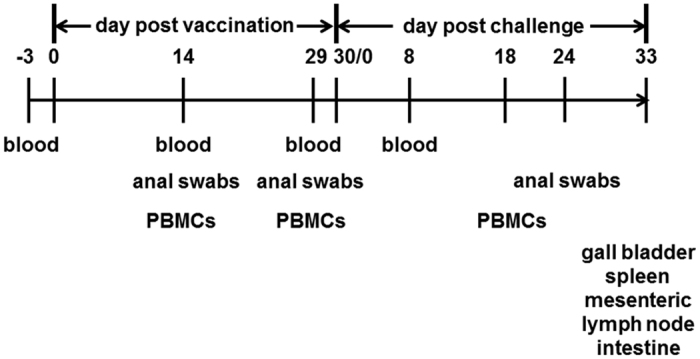
The schematic representation of sample collection from piglets. Piglets were vaccinated with the PTSL vaccine on day 0 and challenged with virulent bacteria on the 30^th^ day post vaccination (=day 0 of challenge). The animals were euthanased on the 33^rd^ day post challenge.

The collection of samples was scheduled as described in Fig. 8. Blood samples were collected from the ear vein of vaccinated piglets 3 days before vaccination, and on the 14^th^ and 29^th^ dpv and and the 8^th^ day dpc. Sera were obtained from the blood samples by centrifugation at 1,700 × g for 10 min at 4 °C. Anal swabs for the analysis of fecal IgA were taken from vaccinated piglets on the 14^th^ and 29^th^ dpv, also on the 24^th^ dpc. About 0.1 g of the anal swab was suspended in 1 ml PBS, and the supernatant was collected after centrifugation at 12,000 × g for 5 min at 4 °C. All samples were stored at −80 °C before analysis.

Peripheral blood mononuclear cells (PBMCs) were extracted from precaval vein from five randomly selected piglets of each group on the 14^th^ and 29^th^ dpv, and from challenged piglets on 18^th^ dpc. Gall bladder, spleen, MLN, intestine sub-parts as duodenum, jejunum, ileum, cecum, and colon were aseptically collected at necropsy. Splenocytes and lymphocytes were isolated from spleen and MLN, respectively. Bile was collected from the gall bladder, centrifuged at 12,000 × g for 5 min at 4 °C and the supernatant was obtained for the analysis of IgA. The intestine segments were rinsed with saline and mucosal scrapes were collected in 1 ml PBS. The supernatant for the analysis of IgA was collected after centrifugation at 12,000 × g for 5 min at 4 °C.

### ELISA

Serum IgG, fecal IgA and intestinal IgA were measured by indirect ELISA as described previously^11^. Inactivated *S.* Choleraesuis antigen was prepared by heating the C500 culture at 80 °C for 10 min in a water bath, and then washed in saline and suspended in PBS to approximately 3 × 10^11^ CFU/ml^15^. The suspension was diluted in carbonate-bicarbonate buffer (pH 9.6) to 1:40 for the detection of IgG and to 1:10 for the detection of IgA. The diluted inactivated *S.* Choleraesuis antigen (100 μl) was added to each well of 96-well ELISA microtiter plates (Nunc, Roskilde, Denmark). The plates were incubated at 4 °C overnight, and then washed 3 times with PBS solution containing 0.05% Tween 20 (Amresco, America). Next, the plates were blocked with 5% skim milk in PBS at 37 °C for 2 h, and then washed three times. Serum samples were 1:80000 diluted in washing buffer containing 2% skim milk, and 100 μl of the solution was added to duplicate antigen-coated wells. For IgA detection, the supernatant of fecal swab, bile and intestinal mucosa was 1:4 diluted and incubated with the antigen in duplicate. The plates were washed three times after incubation for 1 h at 37 °C. Then, 100 μl of 1:2000 diluted HRP labeled anti-pig IgG (H + L) antibody (KPL) or 1:1000 diluted HRP labeled anti-pig IgA antibody was added to the wells, and the plates were incubated at 37 °C for 1 h. Then after 4 washings, a substrate solution containing TMB solutions A and B (Macgene, Beijing, China) was added to the wells for 10 min at room temperature. The reaction was stopped by 50 μl of 2 M sulfuric acid. The plates were read at 450/630 nm using an ELISA reader (Bio-Rad, Richmond, USA).

### Real time RT-PCR

PBMCs collected on the 29^th^ dpv were stimulated with 12 μg/ml of inactivated *S.* Choleraesuis antigen for 12 h. Total RNA was extracted using the Trizol reagent (Invitrogen, USA), and then treated with DNaseΙ (NEB, USA) at 37 °C for 10 min to remove residual genomic DNA. The RNA quantity and purity were determined using UV spectroscopy at OD_260nm_ and OD_280nm_. Finally, 1–2 μg of RNA from each sample was used for transcription to cDNA using the High Capacity cDNA Reverse Transcription Kit (Ambion, Austin, USA).

Cytokines were detected by RT-PCR. The primers for amplifying porcine IL-4, IL-12, and TNF-α were designed by the PerlPrimer software (Table S1). The primers for amplifying porcine GAPDH, IL-2, and IFN-γ have been described previously^40^. The mRNA level of the respective cytokine genes was assessed by RT-PCR performed in the 7500 Real Time PCR System (Ambion, Inc.). The housekeeping gene (GAPDH) and target genes from each sample were amplified in parallel on the same PCR plate programmed to 50 °C for 2 min, 95 °C for 10 min, 40 cycles of 95 °C for 15 s, and 60 °C for 1 min according to the instructions for the SYBR Green PCR master mix (Ambion). Analyses of the relative change (relative to the non-stimulated control) in mRNA expression of target genes was conducted using the 2^−ΔΔCt^ method^41^.

### Intracellular cytokine staining

Porcine PBMCs, splenocytes and lymphocytes were cultured in 12-well microtiter plates (4 × 10^6^ cells/well), and stimulated with 24 μg/ml of inactivated *S.* Choleraesuis antigen for 12 h. All samples were performed in triplicate. The cells were subsequently treated with the BD GolgiStop™ protein transport inhibitor (BD Biosciences, San Diego, CA, USA) for 6 h before the staining. The cells were harvested and suspended in 100 μl PBS containing 10% porcine serum. After washing twice with a cell staining buffer (QuantoBio, Beijing, China), the cells were stained at 4 °C for 30 min, with fluorescein isothiocyanate (FITC)-conjugated anti-pig CD3ε (Southern Biotech, USA) only, or with a combination of FITC-conjugated anti-pig CD3ε (Southern Biotech), biotin-conjugated anti-pig CD4 (Southern Biotech) and Allophycocyanin (APC)-conjugated streptavidin (Southern Biotech), or with a combination of FITC-conjugated anti-pig CD3ε (Southern Biotech) and Spectral Red (SPRD)-conjugated anti-pig CD8 (Southern Biotech). After washing twice with the cell staining buffer, the cells were fixed with 4% paraformaldehyde at 4 °C for 20 min. The fixed cells were washed twice and permeabilized with 250 μl of BD Perm/Wash buffer. Then, the cells were suspended in 50 μl of BD Perm/Wash solution and stained with Phycoerythrin (PE)-conjugated anti-pig IFN-γ (BD ebioscience, USA) at 4 °C for 30 min, followed by two washings. Finally, for each cell sample, at least 20,000 lymphocytes were collected. IFN-γ expressions in gated CD3^+^CD4^+^ cells and CD3^+^CD8^+^ cells were measured using an Accuri™ C6 flow cytometer (BD, USA). For determining the production of IL-2, IFN-γ, and TNF-α in CD3^+^ T cells, the cells were stained with PE-conjugated anti-pig IFN-γ (BD), or APC-conjugated anti-pig IL-2 (R&D Systems, USA), or PE-conjugated Cy5.5 anti-human TNF-α (Biolegend, USA).

### Gross pathology and histopathology examination

Necropsy was performed immediately after the piglets died post-challenge or after euthanasia on the 33^th^ dpc. Liver, spleen, lung, kidney, duodenum, jejunum, ileum, cecum, colon, and rectum tissues were collected and fixed in neutral buffered formalin. The tissues were processed by the routine method for histopathological examination by the Department of Veterinary Pathology, China Agricultural University.

### Data analysis

The data were analyzed using the SPSS statistical package and one-way ANOVA method, followed by the Fisher’s Least Significant Difference test. The study results were expressed as mean ± SD. p values p < 0.05 were considered significant, and p < 0.01 or 0.001 very significant. The analysis of flow cytometry data was performed using the Accuri CFlow software (Accuri Cytometers).

## Additional Information

**How to cite this article**: Zhu, L. *et al*. Mucosal IgA and IFN-γ^+^ CD8 T cell immunity are important in the efficacy of live *Salmonella enteria* serovar Choleraesuis vaccines. *Sci. Rep.* **7**, 46408; doi: 10.1038/srep46408 (2017).

**Publisher's note:** Springer Nature remains neutral with regard to jurisdictional claims in published maps and institutional affiliations.

## Supplementary Material

Supplementary Materials

## Figures and Tables

**Table 1:** Mortality, body weight and *S.* Choleraesuis status of piglets post challenge.

Group	Piglet No.	Survival status	Body weight change (kg)	Isolation of *S.* Choleraesuis			
				liver	lung	spleen	kidney
control	26	Dead	/	^+^	^+^	^+^	^+^
	32	Dead	/	^+^	^+^	^+^	^+^
	33	Alive	−9.9	^+^	^+^	^+^	−
	38	Alive	1.5	−	−	−	−
	50	Dead	/	^+^	^+^	^+^	^+^
PO	13	Alive	13.8	−	−	−	−
	14	Alive*	−	−	−	−	−
	15	Alive	17.8	−	−	−	−
	21	Alive	5.2	−	−	−	−
	23	Alive	24.2	−	−	−	−
IM	1	Dead	/	^+^	^+^	^+^	^+^
	6	Alive	−9.3	^+^	^+^	−	^+^
	7	Alive	13.3	−	−	−	−
	31	Dead	/	−	−	−	−
	36	Alive	9.3	−	−	−	−

/: not determined. ^+^: *Salmonella* was isolated. −: No *Salmonella* was isolated. *The piglet died on the 32^th^ day post challenge, while its body weight increased by 2.4 kg on the 17^th^ day post challenge. Since no bacteria were isolated from this piglet, the pig was regarded as protected by vaccination.
