# Supplementary material for: Mucosal IgA and IFN-γ+ CD8 T cell immunity are important in the efficacy of live Salmonella enteria serovar Choleraesuis vaccines
Source: Sci Rep. 2017 Apr 13;7:46408. doi: 10.1038/srep46408 (PMC5390296; doi:10.1038/srep46408)

**Mucosal IgA and IFN-γ^+^ CD8 T cell immunity are important in the efficacy of live *Salmonella enteria* serovar Choleraesuis vaccines**

Liangquan Zhu^1, 2, †^, Xinxin Zhao^1, 3, †^, Qing Yin^1^, Xianyong Liu^1^, Xiang Chen^4^, Chunjuan Huang^5^ and Xun Suo^1, *^

^1^Key Laboratory of Animal Epidemiology and Zoonosis of Ministry of Agriculture, State Key Laboratory for Agrobiotechnology, National Animal Protozoa Laboratory & College of Veterinary Medicine, China Agricultural University, Beijing, China

^2^China Institute of Veterinary Drug Control, Beijing, China

^3^Institute of Preventive Veterinary Medicine, Sichuan Agricultural University, Chengdu, Sichuan, China

^4^Jiangsu Key Laboratory of Zoonosis, Yangzhou University, Yangzhou, Jiangsu, China

^5^Institute of Biophysics, Chinese Academy of Sciences, Beijing, China

^†^ These authors contributed equally to this work.

* Correspondence and requests for materials should be addressed to X.S. ([suoxun@cau.edu.cn](mailto:suoxun@cau.edu.cn))

**Supporting Information**

**Table S1. Primers used in the RT-PCR.**

| Gene | Primer sequence | Product size (bp) | Efficiency (%) |
| --- | --- | --- | --- |
| IL-4 | 5’-GGCAAACATGACCTGTTCTG-3’  5’-CCTTCATAATCGTCTTTAGCCT-3’ | 83 | 95 |
| IL-12 | 5’- CAGGGACATCATCAAACCAG -3’  5’- TGAACACCAAACATCAGGGA -3’ | 141 | 98 |
| TNF-α | 5’- CCCAGAAGGAAGAGTTTCCA -3’  5’- TTTGACATTGGCTACAACGTG -3’ | 116 | 102 |

**Figure S1. The PTSL vaccine elicited high levels of serum antibody responses in piglets.** Each randomly selected twenty piglets of the same condition were vaccinated with the PTSL vaccine with 1/2×, 1× or 2× the standard vaccine dose via oral and intramuscular administration. Serum was obtained on the 14^th^ day post vaccination, and the IgG level was determined by indirect ELISA. *, p<0.05, **, p<0.01, ***, p<0.001.


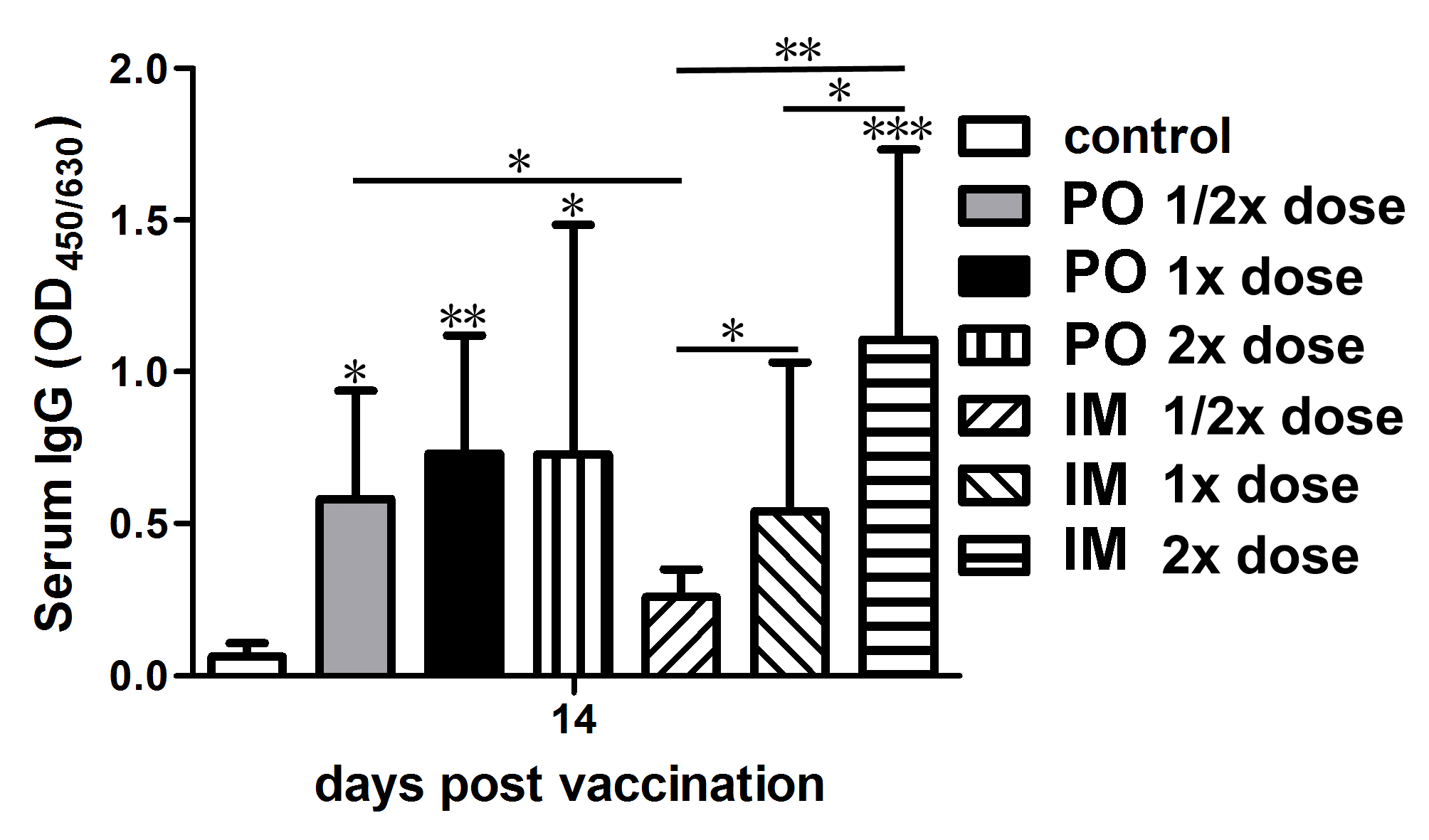

Supplement: Supplementary Materials [file srep46408-s1.doc]
